# Transcriptomic profiling of pancreatic alpha, beta and delta cell populations identifies delta cells as a principal target for ghrelin in mouse islets

**DOI:** 10.1007/s00125-016-4033-1

**Published:** 2016-07-07

**Authors:** Alice E. Adriaenssens, Berit Svendsen, Brian Y. H. Lam, Giles S. H. Yeo, Jens J. Holst, Frank Reimann, Fiona M. Gribble

**Affiliations:** 1 Metabolic Research Laboratories, Wellcome Trust–MRC Institute of Metabolic Science, Addenbrooke’s Hospital, Cambridge, CB2 0QQ UK; 2 Novo Nordisk Foundation Center for Basic Metabolic Research, University of Copenhagen, Copenhagen, Denmark; 3 Department of Biomedical Sciences, Faculty of Health and Medical Sciences, University of Copenhagen, Copenhagen, Denmark

**Keywords:** Alpha cells, Beta cells, Delta cells, Ghrelin, Glucagon, Insulin, RNA sequencing, Somatostatin

## Abstract

**Aims/hypothesis:**

Intra-islet and gut–islet crosstalk are critical in orchestrating basal and postprandial metabolism. The aim of this study was to identify regulatory proteins and receptors underlying somatostatin secretion though the use of transcriptomic comparison of purified murine alpha, beta and delta cells.

**Methods:**

*Sst-Cre* mice crossed with fluorescent reporters were used to identify delta cells, while *Glu-Venus* (with Venus reported under the control of the *Glu* [also known as *Gcg*] promoter) mice were used to identify alpha and beta cells. Alpha, beta and delta cells were purified using flow cytometry and analysed by RNA sequencing. The role of the ghrelin receptor was validated by imaging delta cell calcium concentrations using islets with delta cell restricted expression of the calcium reporter GCaMP3, and in perfused mouse pancreases.

**Results:**

A database was constructed of all genes expressed in alpha, beta and delta cells. The gene encoding the ghrelin receptor, *Ghsr*, was highlighted as being highly expressed and enriched in delta cells. Activation of the ghrelin receptor raised cytosolic calcium levels in primary pancreatic delta cells and enhanced somatostatin secretion in perfused pancreases, correlating with a decrease in insulin and glucagon release. The inhibition of insulin secretion by ghrelin was prevented by somatostatin receptor antagonism.

**Conclusions/interpretation:**

Our transcriptomic database of genes expressed in the principal islet cell populations will facilitate rational drug design to target specific islet cell types. The present study indicates that ghrelin acts specifically on delta cells within pancreatic islets to elicit somatostatin secretion, which in turn inhibits insulin and glucagon release. This highlights a potential role for ghrelin in the control of glucose metabolism.

**Electronic supplementary material:**

The online version of this article (doi:10.1007/s00125-016-4033-1) contains peer-reviewed but unedited supplementary material, which is available to authorised users.

## Introduction

The pancreatic islets provide a centre where signals indicating the nutritional status of the body, including factors such as enteroendocrine hormones, nutrients, metabolites and neuronal signals, can converge and initiate changes in pancreatic hormone secretion to regulate blood glucose levels. Insulin (released from beta cells) and glucagon (released from alpha cells) exert opposite effects on glycaemia, with insulin promoting glucose uptake in conditions of high glucose and glucagon initiating hepatic glucose production in periods of decreasing glucose levels [1]. Nuanced interactions and crosstalk between islet cell types are critical in maintaining tight control over blood glucose equilibrium, and elucidating the ways in which enteric signals and islet cells interact to influence circulating glucose levels could provide insights into the mechanisms underlying altered glycaemic control and diabetes [2, 3].

A key paracrine mediator within islet cells is somatostatin (SST), which is produced by pancreatic delta cells. SST appears to exert tonic suppression of insulin and glucagon release within islets [4]. The importance of this potent paracrine mechanism is illustrated by experiments showing that whole-animal genetic ablation of *Sst* results in aberrant secretion of both insulin and glucagon from isolated islets in response to glucose [5]. Indeed, the dysregulation of SST-mediated negative-feedback loops has been implicated in the development of type 2 diabetes [6]. Compared with our knowledge of insulin and glucagon release, there is still much to learn about the regulatory pathways and cellular machinery underlying SST secretion. Identifying how delta cells differ from their neighbouring alpha and beta cells is crucial for interpreting transcriptomic and functional data obtained from whole islets [7].

Ghrelin is a peptide hormone that has been identified as a key component of the gut–brain axis [8]. It is synthesised predominantly in the stomach [9, 10] and gastrointestinal tract [11], although there have been reports of ghrelin-producing epsilon cells in adult islets [12, 13]. Ghrelin levels in plasma are influenced by nutritional status and may influence growth hormone secretion, appetite and fat deposition [14]. Importantly, there are indications that ghrelin plays a role in the regulation of the pancreas in response to changes in glucose levels [15]. A large number of reports have examined the effects of the active acylated form of ghrelin on glucose-stimulated insulin secretion. The consensus of these studies is that ghrelin exerts acute inhibition of insulin release [16–19], and that ghrelin infusions lead to impaired glucose tolerance [20, 21]. In addition, pharmacological inhibition of ghrelin acylation (which is essential for the biological activity of ghrelin) via blockade of ghrelin *O*-acyltransferase results in significant increases in glucose-stimulated insulin secretion and improves overall glucose tolerance [22].

The cognate receptor for ghrelin is the growth hormone secretagogue receptor (GHSR) [9]. The effects of ghrelin on insulin release are purportedly through direct receptor-mediated modulation of beta cell activity [23, 24]. However, the predominant G_αq_ coupling of GHSR [25] and the insulinostatic effects of ghrelin, if indeed mediated directly via beta cells, are paradoxical. Because of the therapeutic potential of manipulating the ghrelin axis in individuals with obesity and diabetes [26], the mechanism by which ghrelin inhibits insulin release warrants further exploration.

The aims of this study were to build a transcriptomic profile of pancreatic delta cells, in comparison with alpha and beta cells, and to identify specific delta cell markers and regulators. Having demonstrated *Ghsr* expression to be highly enriched in delta cells, we further aimed to characterise the effects of ghrelin on delta cell signalling pathways and islet cell secretory profiles.

## Methods

### Solutions

Unless otherwise stated, all chemicals were obtained from Sigma-Aldrich (Poole, UK). The standard bath solution contained 138 mmol/l NaCl, 4.5 mmol/l KCl, 4.2 mmol/l NaHCO_3_, 1.2 mmol/l NaH_2_PO_4_, 2.6 mmol/l CaCl_2_, 1.2 mmol/l MgCl_2_ and 10 mmol/l HEPES (pH 7.4, NaOH). Mouse ghrelin and SST receptor (SSTR) antagonists (H-5884 + H-6056) were obtained from Bachem (Bubendorf, Switzerland).

### Animals

All animal procedures were approved by the local ethics committee and conformed to UK Home Office regulations or those of the Animal Experiments Inspectorate, Ministry of Justice, Denmark, and the eighth edition of the Guide for the Care and Use of Laboratory Animals (2011) (http://grants.nih.gov/grants/olaw/guide-for-the-care-and-use-of-laboratory-animals.pdf). For the isolation of purified populations of alpha and beta cells, transgenic mice expressing the Venus fluorophore under the control of the proglucagon promoter, *Glu* (also known as *Gcg*), (*Glu-Venus*) [27] were used. For the introduction of delta cell specific transgenes for FACS-mediated purification or the introduction of the genetically encoded calcium sensor GCaMP3 [28], transgenic mice expressing *Cre* under the control of the *Sst* promoter [29, 30] were used. These mice were crossed with reporter strains containing genes encoding tandem red fluorescent protein (*tdRFP*) (a gift from H. J. Fehling, University Clinic Ulm, Ulm, Germany), *GCaMP3* (Charles River, Margate, UK) or enhanced yellow fluorescent protein (*EYFP*) (Charles River) in the *Rosa26* locus [28, 31]. All mice were on a C57BL/6 background.

### Perfused mouse pancreases

Male C57BL/6 J mice (age approximately 10 weeks, purchased from Taconic, Ejby, Lille Skensved, Denmark) were anaesthetised and pancreases were isolated and perfused in situ as described previously [32]. Pancreases were perfused with a modified Krebs Ringer bicarbonate buffer containing, in addition, 5% dextran (Dextran Products, Toronto, ON, Canada), 0.1% BSA, fumarate, glutamate and pyruvate (5 mmol/l of each) and 12 mmol/l glucose. Test substances included mouse ghrelin (1–100 nmol/l), SSTR antagonists (1 μmol/l) and arginine (10 mmol/l). Hormone concentrations were measured using in-house RIA [33–35].

### Islet isolation and FACS

Transgenic mice expressing the fluorescent protein Venus under the control of the proglucagon promoter (*Glu-Venus*) or EYFP under the control of the *Sst* promoter (*Sst-Cre*/*Rosa26*^*EYFP*^) were killed and the pancreases were injected with collagenase V (0.5 mg/ml). Pancreases were digested at 37°C. Islets were hand-picked into HBSS containing 0.1% wt/vol. fatty acid-free BSA. Each pancreas yielded approximately 150–300 islets. Islets from two to five mice were pooled for each replicate sample. Islets were disrupted into single cells by trituration following incubation in Ca^2+^-free HBSS containing 0.1× trypsin/EDTA. Cells were sorted by flow cytometry using a BD Influx cell sorter (BD Biosciences, San Jose, CA, USA) equipped with a 488 nm laser for excitation of Venus and EYFP. Venus-negative cells from the *Glu-Venus* sorts were further subdivided to collect a population with high side and forward scatter and high background autofluorescence at 530 and 580 nm to isolate beta cells. Cells were collected into RLT lysis buffer (Qiagen, Manchester, UK) and frozen on dry ice.

### RNA extraction and quantitative RT-PCR

Total RNA was extracted using an RNeasy Micro kit (Qiagen) according to the manufacturer’s protocol. Quantitative (q)RT-PCR was performed with a 7900 HT Fast Real-Time PCR system (Applied Biosystems, Warrington, UK). The PCR reaction mix consisted of approximately 20 ng first-strand cDNA template, 6-carboxyfluorescein/quencher probe/primer mixes (Thermo Fisher Scientific, Loughborough, UK) and PCR Master Mix (Thermo Fisher Scientific), and was amplified for 40 cycles. Samples where target gene expression was undetected were assigned C_t_ values of 40. Expression of the selected targets was compared with that of *Actb*, measured on the same sample in parallel on the same plate, giving a C_t_ difference (ΔC_t_). Mean and SEM calculations and statistical analyses were performed on the ΔC_t_ data and only converted to relative expression levels (${2}^{\Delta {\mathrm{C}}_{\mathrm{t}}}$) for presentation in the figures.

### RNA sequencing

Total RNA was extracted using an RNeasy Plus Micro kit (Qiagen) according to the manufacturer’s instructions. The quality of the extracted RNA was checked using a Bioanalyzer RNA Pico kit (Agilent Technologies, Stockport, UK), indicating RIN (RNA Integrity Number) values between 7.2 and 9.4. RNA was amplified using the Ovation RNA sequencing RNA-Seq System V2 (NuGEN Technologies, Leek, the Netherlands) (six replicates were used for delta cells, five for alpha cells and four for beta cells, totalling 15 samples). An RNA sequencing library was prepared using the Ovation Rapid DR Library System (NuGEN) and sequenced using an Illumina HiSeq 2500 system at the Genomics Core Facility, Cancer Research UK Cambridge Institute (Cambridge, UK).

### Islet isolation for imaging experiments

Transgenic mice expressing the genetically encoded calcium sensor GCaMP3 under the control of the *Sst* promoter (*Sst-Cre*/*Rosa26*^*tdRFP/GCaMP3*^) were killed and their islets were isolated, dissociated into cell clusters and plated onto Matrigel-coated glass-bottom dishes. Cells were incubated at 37°C and 5% CO_2_ in RPMI 1640 medium containing 11.1 mmol/l glucose and 10% FCS vol./vol. Cells were imaged 24–48 h after plating.

### Calcium imaging

Pancreatic delta cells were imaged 1–2 days after plating. GCaMP3-positive cells were imaged and data recorded as previously described [29]. All bath solutions contained 1 mmol/l glucose. Average fluorescence intensities were calculated over 10 s time windows for the entirety of the experiment. GCaMP3 intensity values over the entire trace for each experiment were normalised to the absolute baseline, which was calculated by taking the average GCaMP3 intensity values over two 1 min intervals at the beginning and end of the experiment when cells were in basal conditions. Responses to test reagents were calculated by determining the average normalised GCaMP3 intensity over a 2 min interval during perfusion of the test reagent divided by the average normalised GCaMP3 intensity over a 2 min interval taken before application of the test reagent to give a fold-change value. Cells were included in the analysis if they responded to 30 mmol/l KCl (*n* = 74); *n* = 19 cells were excluded from the analysis due to spontaneous and erratic GCaMP3 intensity fluctuations.

### Data analysis

All statistical analyses were conducted using Microsoft Excel and GraphPad Prism 5.0 (GraphPad Software, La Jolla, CA, USA). Statistical significance was calculated using a Student’s single-sample or two-sample *t* test or via ANOVA with either a Tukey, Dunnett’s or Bonferroni post hoc test, as appropriate. The threshold for significance was set at *p* < 0.05. Sequence reads were demultiplexed using the CASAVA pipeline (Illumina, Little Chesterford, UK) and then aligned to the mouse genome (GRCm38) using TopHat version 2.0.11 (http://ccb.jhu.edu/software/tophat/index.shtml). Raw read counts and fragments per kilobase of transcript per million mapped reads (FPKM) were generated using Cufflinks version 2.2.1 (http://cole-trapnell-lab.github.io/cufflinks) and differential gene expression was determined using edgeR (www.bioconductor.org/packages/release/bioc/html/edgeR.html).

## Results

### Transcriptomic profiling of isolated populations of islet cells revealed key identifiers of each cell type

**Fig. 1:**
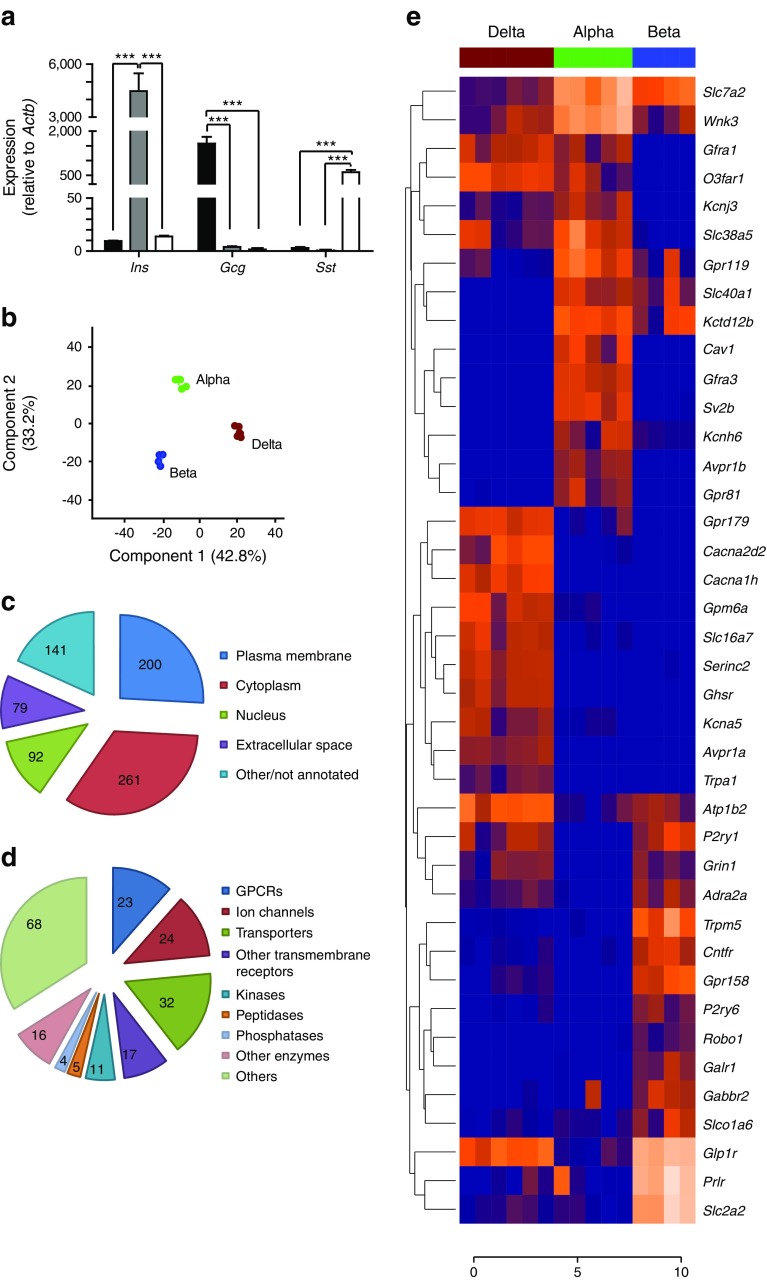
Transcriptomic profiling of pancreatic alpha, beta and delta cells. RNA was extracted from purified populations of alpha, beta and delta cells, and converted to cDNA or prepped for RNA sequencing. (**a**) Populations of alpha (black bars), beta (grey bars) and delta (white bars) cells were checked for *Ins*, *Gcg* and *Sst* enrichment, respectively, using qPCR analysis. Data are presented as the geometric mean, with error bars (SEM) calculated from log_2_ data. Each column represents the average expression from three separate samples. Significance comparisons were calculated by one-way ANOVA with Bonferroni post hoc comparison; ***p < 0.001. (**b**) RNA from five alpha cell samples, four beta cell samples and six delta cell samples was sequenced using SE50 sequencing. Differential gene expression was determined using edgeR, and a principle component analysis plot was constructed using a false discovery rate of 5% and a sensitivity threshold of FPKM values >1. (**c**) Pie chart showing the cellular distribution of genes differentially expressed between islet cell types. (**d**) Pie chart showing the distribution of differentially expressed genes found at the plasma membrane. (**e**) Heatmap showing the top 40 most differentially expressed genes found at the plasma membrane. Data are presented as log_2_ FPKM

We separated populations of alpha and beta cells via FACS from *Glu-Venus* mice and populations of delta cells from *Sst-Cre*/*Rosa26*^*EYFP*^ mice. Quantitative PCR (qPCR) analysis of the relative expression of *Ins*, *Gcg* and *Sst* in cDNA isolated from these purified populations of islet cells confirmed the enrichment of *Ins* in beta cells, *Gcg* in alpha cells and *Sst* in delta cells (Fig. 1a).

We next performed RNA sequencing analysis of these isolated populations of alpha, beta and delta cells to build a transcriptomic profile for each cell type. The mapping efficiency was 82.8%. Principal component analysis revealed that alpha, beta and delta cells clustered separately, indicating that they differ considerably in their gene-expression profiles. Using a false discovery rate of 5% and a lower sensitivity threshold of 1 FPKM, we identified 773 genes that were differentially expressed between alpha, beta or delta cells (Fig. 1b, electronic supplementary material [ESM] Table 1), of which 200 genes encoded proteins expressed on the plasma membrane (Fig. 1c). Of the cell surface markers, we found that 23 G-protein coupled receptors (GPCRs), 24 ion channels and 32 membrane transporters were differentially expressed between alpha, beta and delta cells (Fig. 1d). The top 40 cell surface markers for each cell type are depicted as a heatmap in Fig. 1e, and details of all 773 differentially expressed genes are given in ESM Table 1.

**Fig. 2:**
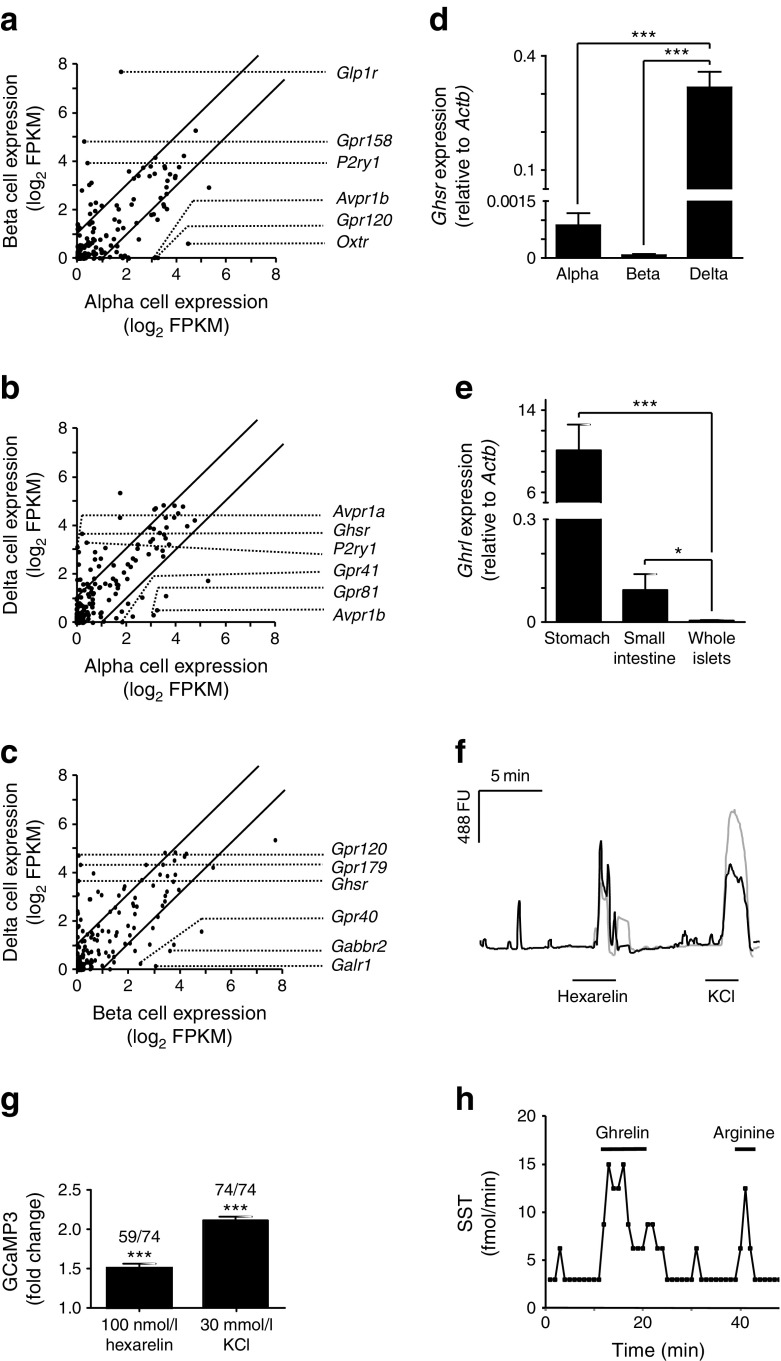
Confirmation of *Ghsr* expression and GHSR activity in delta cells. (**a**–**c**) Log_2_ of FPKM values for GPCRs expressed by each cell type were plotted against each other: (**a**) alpha vs beta cells; (**b**) alpha vs delta cells; and (**c**) beta vs delta cells. A threshold of twofold differential expression was set. Three of the most highly enriched and expressed GPCRs for each cell type are indicated on the graphs. (**d**, **e**) Histograms showing the relative expression of *Ghsr* in pancreatic alpha, beta and delta cells (**d**) and *Ghrl* in the whole stomach, small intestine and islets (**e**). Expression was analysed by qPCR and compared with that of *Actb* in the same sample. Data are presented as the geometric mean, with error bars (SEM) calculated from log_2_ data. Each column represents the average expression from three separate samples. Two to five mice were pooled for each sample in (**d**) and one mouse was used for each sample in (**e**). Significance comparisons were calculated by one-way ANOVA with Bonferroni post hoc comparison; *p < 0.05, ***p < 0.001. (**f**, **g**) Pancreatic islets from *Sst-Cre*/*Rosa26* ^*tdRFP/GCaMP3*^ mice were dispersed and cultured on glass-bottom dishes and imaged 24–48 h after plating. Delta cells were excited with 488/8 nm, and the GCaMP3 fluorescence (488 fluorescence units [FU]) was recorded. Cells were perfused with either 100 nmol/l hexarelin or 30 mmol/l KCl, as indicated. Representative responses of two delta cells monitored in parallel in the same dish are shown in black and grey (**f**). Mean changes in GCaMP3 in cells from seven mice are shown in a histogram (**g**), with the number of responding cells out of the total number of cells imaged for each condition shown above each bar. Data represent the mean ± SEM of the number of responding cells. Significance above baseline was calculated using a single Student’s *t* test; ***p < 0.001. (**h**) A whole pancreas was perfused with 3.5 mmol/l glucose and treated with 10 nmol/l ghrelin and 10 mmol/l arginine, as indicated, and SST concentrations were measured every minute

To identify the key regulatory receptors for each cell type, we plotted the expression levels of *Gpcr*s in alpha vs beta cells, alpha vs delta cells and beta vs delta cells (Fig. 2a–c). *Gpcr*s specific to alpha, beta or delta cells were identified using a cut-off of twofold differential expression. *Ghsr* was one of the most highly enriched *Gpcr*s found in delta cells compared with both alpha and beta cells, and its enrichment was confirmed by qPCR (Fig. 2d). *Ghsr* was undetectable in beta cells and very lowly expressed in alpha cells (alpha vs delta cells, *p* < 0.001; beta vs delta cells *p* < 0.001; alpha vs beta cells *p* < 0.01).

To examine candidate sites of ghrelin production in the mouse, we measured the expression of ghrelin (*Ghrl*) in whole islets, the whole stomach and the whole small intestine by qPCR (Fig. 2e). *Ghrl* expression in whole islets was low and only detected at an average cycle threshold of 36.4, substantially lower than in the stomach and small intestine (*p* < 0.001 and *p* < 0.05, respectively). We were also unable to detect acyl-ghrelin in perfusates from adult mouse pancreases perfused at 3.5 mmol/l glucose, using a ghrelin assay with a working limit of detection of approximately 25 pg/ml (data not shown).

### Calcium imaging in dispersed islets revealed GHSR-mediated activation of delta cells

To establish the functional role of GHSR activation in delta cells, we performed calcium imaging experiments in dispersed islet cultures from *Sst-Cre*/*Rosa26*^*tdRFP/GCaMP3*^ mice. Delta cells were identified by their tdRFP expression, and their calcium responses to 100 nmol/l hexarelin, a stable ghrelin analogue, were recorded in real time using the genetically encoded calcium indicator GCaMP3. Hexarelin elicited significant increases in GCaMP3 emission, indicative of an increased cytosolic calcium concentration, in 59 out of 74 delta cells (1.51-fold increase in GCaMP3 fluorescence, *p* < 0.001; Fig. 2f,g).

### Ghrelin inhibited insulin and glucagon secretion in an SST-dependent manner

**Fig. 3:**
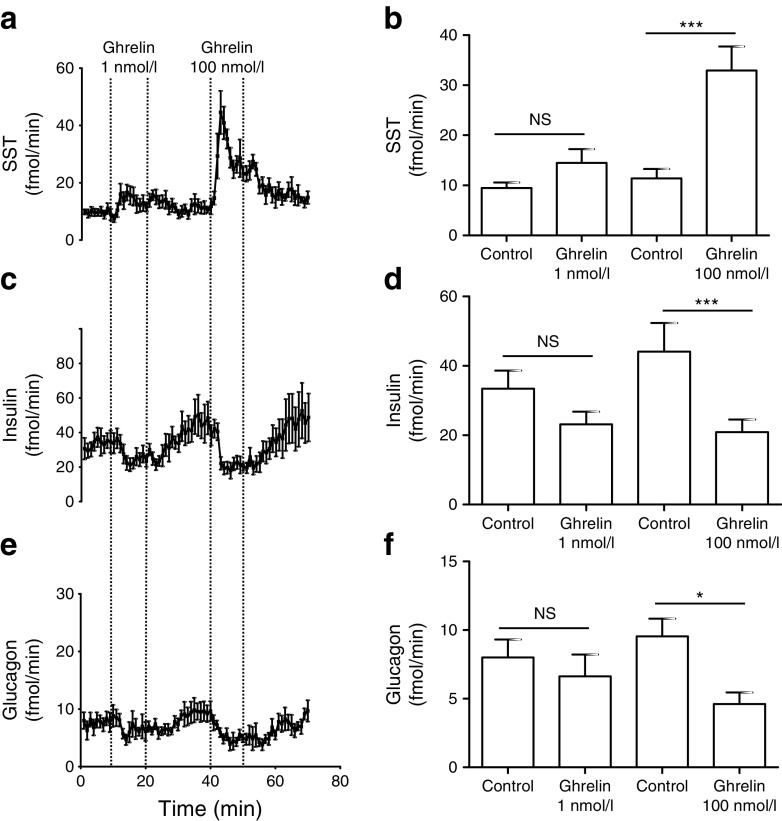
Ghrelin stimulated SST release, while decreasing insulin and glucagon release, in a perfused pancreas model. Whole pancreases were perfused with 12 mmol/l glucose (control) and treated with 1 or 100 nmol/l ghrelin, and the secretion of SST (**a**, **b**), insulin (**c**, **d**) and glucagon (**e**, **f**) was measured. Mean hormone outputs were averaged over 5 min before addition of the test substance and during the final 5 min of test substance perfusion. Data are represented as means ± SEM. Significance was tested by one-way ANOVA with post hoc Tukey modification; *n* = 7; *p < 0.05, ***p < 0.001

To establish a physiological role for delta cell specific activation by GHSR binding, we examined the effects of ghrelin on insulin, glucagon and SST secretion in a perfused mouse pancreas model. In a pilot experiment, 10 nmol/l ghrelin increased SST secretion in a pancreas perfused with 3.5 mmol/l glucose (Fig. 2h), consistent with the finding that hexarelin increased delta cell Ca^2+^ even at low glucose concentrations. In further experiments, pancreases were perfused with 1 or 100 nmol/l ghrelin in the presence of 12 mmol/l glucose to enable concurrent assessment of a potential inhibitory effect on insulin secretion. Both 1 and 100 nmol/l ghrelin evoked immediate increases in SST release (1.6- and 2.7-fold, respectively; Fig. 3a,b). These increases in SST coincided with concomitant decreases in the secretion of insulin (1.4- and 2.1-fold; Fig. 3c,d) and glucagon (1.1- and 2.1-fold; Fig. 3e,f).

**Fig. 4:**
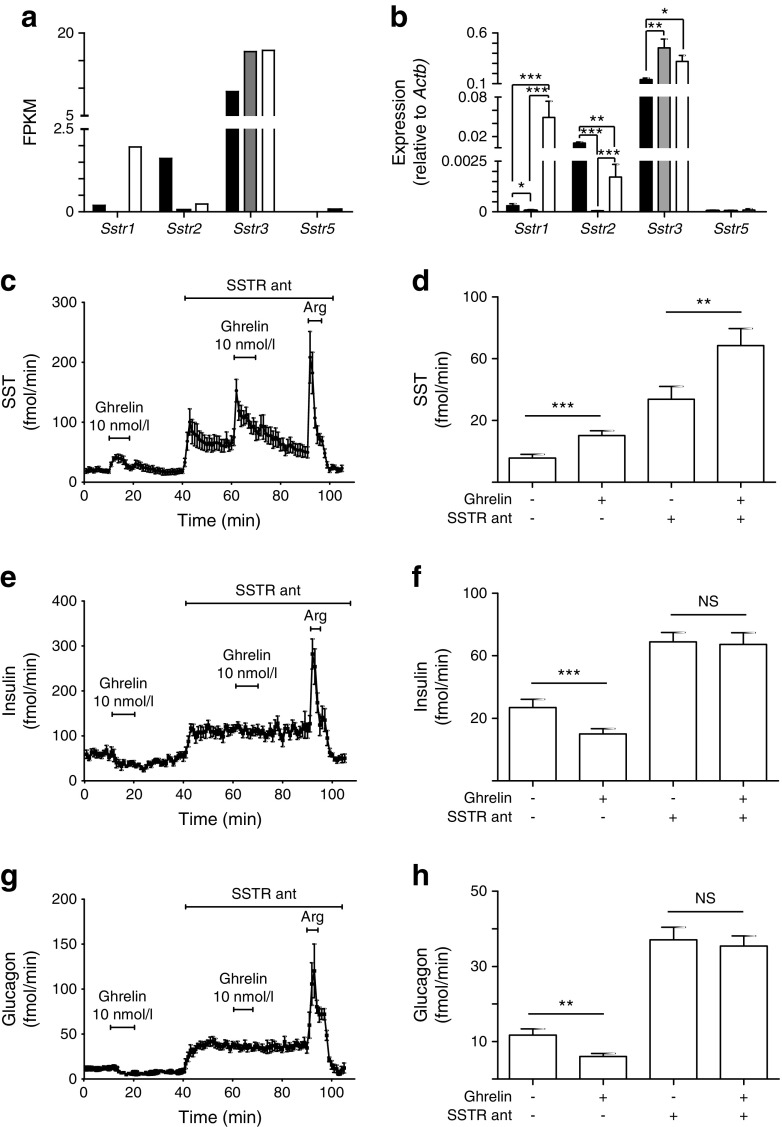
The effects of ghrelin on SST, insulin and glucagon were sensitive to SSTR. (**a**) Expression levels of *Sstr* in alpha (black bars), beta (grey bars) and delta (white bars) cells, as determined by RNA sequencing, were plotted and their expression was confirmed by qPCR (**b**). Data are presented as the geometric mean, with error bars (SEM) calculated from log_2_ data. Each column represents the average expression from three separate samples. Two to six mice were pooled for each sample in (**a**) and one mouse was used for each sample in (**b**). Significance comparisons were calculated by one-way ANOVA with Bonferroni post hoc comparison; *p < 0.05, **p < 0.01, ***p < 0.001. (**c**, **d**) Whole perfused pancreases were perfused with 12 mmol/l glucose (basal) and treated with 10 nmol/l ghrelin in the presence and absence of the SSTR inhibitors H6056 and H5884 (SSTR ant), and the secretion of SST (**c**, **d**), insulin (**e**, **f**) and glucagon (**g**, **h**) was measured; 10 mmol/l arginine (Arg) was used as a positive control. Mean hormone outputs were averaged over 5 min before addition of the test substance and during the final 5 min of test substance perfusion. Data are represented as means ± SEM. Significance was tested by one-way ANOVA and paired Student’s *t* test; *n* = 8; **p < 0.01, ***p < 0.001

As beta cells express negligible levels of *Ghsr*, and alpha cells express significantly lower levels of *Ghsr* compared with delta cells, we hypothesised that the effects of ghrelin on insulin and glucagon secretion were mediated by SST. Examination of our RNA sequencing database revealed that the most prominent *Sstr* expressed in beta cells was *Sstr3*, whereas alpha cells expressed both *Sstr2* and *Sstr3* (Fig. 4a). In the RNA sequencing analysis, *Sstr5* was undetectable in all alpha and beta cell samples, but gave a low signal in one out of six delta cell samples, whereas *Sstr4* was undetectable in all samples analysed. Expression levels of *Sstr1*, *Sstr2*, *Sstr3* and *Sstr5* were further quantified by qPCR (Fig. 4b). Perfusion experiments were repeated, and 10 nmol/l ghrelin was applied with and without the combination of the SSTR antagonists H6056 and H5884 (both 1 μmol/l), which are inhibitors of SSTR2, SSTR3 and SSTR5. Addition of the SSTR inhibitor cocktail to the perfusate containing 12 mmol/l glucose increased the secretion of SST (2.6-fold; *p* < 0.01), insulin (twofold, *p* < 0.001) and glucagon (threefold, *p* < 0.001). In the continued presence of the antagonist cocktail, the ghrelin infusion still stimulated SST secretion, but its effects on insulin and glucagon release were abolished (Fig. 4c–h).

## Discussion

In this study, we identified the transcriptome of pancreatic delta cells and performed a comparative transcriptomic analysis with beta and alpha cells. Amongst islet-expressed *Gpcr*s, we found *Ghsr* to be significantly enriched in delta cells over both alpha and beta cells. Although we were unable to identify antibodies suitable for confirming GHSR localisation at the protein level, the functional relevance of this receptor in delta cells was confirmed by the finding that GHSR agonism elicited increases in cytosolic calcium levels in isolated delta cells, and that in the perfused pancreas, ghrelin stimulated SST release while attenuating insulin and glucagon release in an SSTR-sensitive manner.

A multitude of studies involving genetic and pharmacological manipulation of GHSR have concluded that the action of ghrelin on glucose tolerance and glycaemia is reliant on GHSR binding and that its blockade, even on a high-fat background, improves glucose handling [12, 16, 36–38]. Previous studies have concluded that the effects of ghrelin on insulin secretion are mediated by its direct binding to GHSR located on the beta cell plasma membrane, but the underlying signalling mechanism is difficult to explain. GHSR is predominantly G_αq_ coupled, so, like other beta cell G_αq_-coupled receptors such as the muscarinic receptor M3, its activation would be predicted to enhance rather than inhibit insulin secretion. However, GHSR blockade in isolated islets has been reported to increase insulin release and cytosolic calcium in beta cells via a pertussis toxin-sensitive pathway [16], implicating G_i/o_ G proteins [39], and to be impaired by antisense oligonucleotides against G_αi2_ [23]. Administration of pertussis toxin has also been reported to render ghrelin incapable of lowering plasma insulin levels in vivo [23].

To account for the paradoxical G_i/o_ dependence of a response downstream of a G_αq_-coupled receptor, some have suggested non-canonical coupling of the ghrelin receptor to G_i/o_ G proteins via recruitment and heteromerisation of GHSR with SSTR5 in beta cell lines [24]. However, our transcriptomic analysis found negligible expression of *Ghsr* and *Sstr5* in mouse beta cells. This is unlikely to reflect technical limitations, as we have previously been able to detect *Sstr2*, *Sstr3* and *Sstr5* in intestinal L cells [40]. In the context of pancreatic islets, our data confirm relatively specific expression of *Sstr2* in alpha cells, but the high expression of *Sstr3* was unexpected [41], suggesting that conclusions based on SSTR-selective agents and antibodies should be revisited. A recent study reported that re-expression of *Ghsr* specifically in beta cells on a *Ghsr*^–/–^ background rescued the ability of a GHSR antagonist to enhance glucose-stimulated insulin release during a glucose tolerance test [42], supporting the direct detection of ghrelin by beta cells and suggesting that even extremely low levels of *Ghsr* expression might modulate beta cell activity. To explain the GHSR-mediated suppression of insulin release and the involvement of a G_i/o_-dependent pathway, our findings alternatively suggest that the inhibitory effect of ghrelin on insulin release is not entirely mediated directly via the beta cell, but instead proceeds at least in part by the activation of GHSR on delta cells, triggering SST release that subsequently inhibits beta cells through SSTR activation. Similar conclusions were reached in a paper submitted while this manuscript was under review [43].

Our work presents an exhaustive transcriptomic comparison between murine pancreatic alpha, beta and delta cells (available at www.ncbi.nlm.nih.gov/geo), providing a database for identifying factors that similarly or uniquely regulate different islet cell types. The transgenes used to fluorescently label alpha and delta cells did not alter islet architecture or the relative proportions of islet cell types [30], but we cannot rule out the possibility that they had subtle effects on gene expression. Several recent studies have similarly analysed the gene-expression profiles of pancreatic alpha and beta cells [44–47], but this, together with the study conducted in parallel by DiGruccio et al [43], is the first study to compare delta cells with neighbouring alpha and beta cells. Delta cells exert a tonic inhibitory tone over both insulin and glucagon release, as evident from the elevated rates of basal insulin and glucagon release from perfused pancreases in the presence of SSTR inhibitors. Whether and how agonists/antagonists modulate SST signalling will therefore be an important consideration in the design of new antidiabetic drug targets, as well as for our understanding of the endocrine and metabolic control of insulin secretion.

## Electronic supplementary material

Below is the link to the electronic supplementary material.

ESM Table 1 (XLSX 72 kb)

## Abbreviations

EYFP: Enhanced yellow fluorescent protein
FPKM: Fragments per kilobase of transcript per million mapped reads
GHSR: Growth hormone secretagogue receptor
GPCR: G-protein coupled receptor
qPCR: Quantitative PCR
*Sst*/SST: Somatostatin
SSTR: Somatostatin receptor
tdRFP: Tandem red fluorescent protein
